# Supplementary material for: Opinion Polarization in Human Communities Can Emerge as a Natural Consequence of Beliefs Being Interrelated
Source: Entropy (Basel). 2022 Sep 19;24(9):1320. doi: 10.3390/e24091320 (PMC9498196; doi:10.3390/e24091320)
Supplement: Supplementary file 1 [file entropy-24-01320-s001.zip › entropy-1848838-supplementary.pdf]

Supporting Information for the Manuscript Entitled "Opinion Polarization in Human Communities Can Emerge As a Natural Consequence of Beliefs Being Interrelated"

The aim of the present Supplementary Information is to study the robustness and statistical behaviour of the results presented in the main text. The first subsection provides an analysis of the statistical behaviour of the simulation, while in the second part the effects of the parameters are surveyed.

Statistical Behaviour

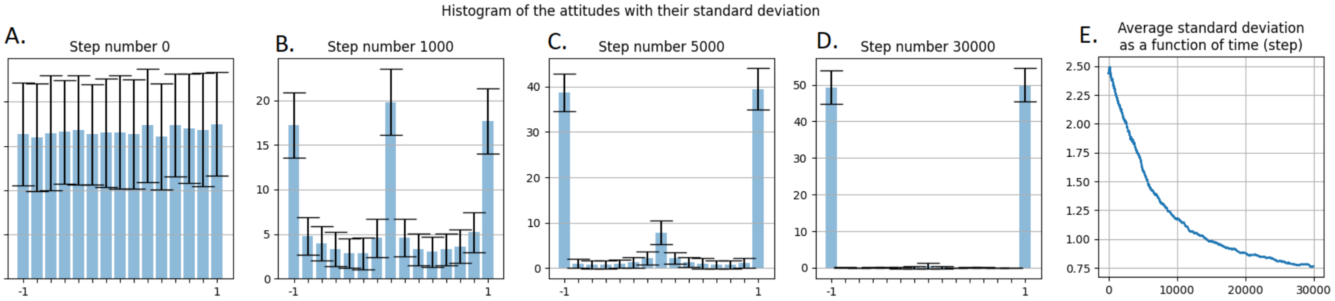

Figure S1: Histogram of the attitude values, along with their standard deviations, for four time-steps: $t = 0$ (sub-figure A), $t = 1,000$ (B), $t = 5,000$ (C), and $t = 30,000$ (D). Sub-figure E depicts the average of the standard deviations along the entire run, from $t = 0$ to $t = 30,000$. As it can be seen, with the growth of t, the standard deviations of the histograms values decrease, leading to the conclusion that the long-term outcome of the simulation – basically: its predictions – are independent of the initial values. These histograms can be considered as slices of the 3D histograms presented in various places throughout the manuscript (for example in sub-figures A and B in Figure 2 in the main text), and the Supporting Information. The parameters are: number of runs: 200. Number of bins: 15. Number of iterations: 30,000. Initial distribution: uniform, taking values from the [-1, 1] interval. Number of agents, $N = 100$. Time-steps: $t = 0$ (sub-figure A), $t = 1,000$ (sub-figure B), $t = 5,000$ (sub-figure C) and $t = 30,000$ (sub-figure D).

In the initial step of the simulation, the attitude values are set according to a certain distribution. In the results presented in the main text, the initial distribution is uniform, taking values from the $[-1, 1]$ interval. This setting calls for two questions: (i) How does the dynamics alter in case the initial distribution is not uniform?, and (ii) How does the stochastic nature (originating from the random numbers) affect the simulation results? In order to answer the first question, we have run the simulation with various types of initial distribution. The results are summarized in Figure S6 in the subsection entitled "The initial distribution of the attitude values".

In order to answer the second question, we have executed the simulation 200 times and calculated the histogram of the attitude values, along with their standard deviation, at each time-step t. Sub-figures A to D in Figure S1 shows the results for four time-steps: the initial one: $t = 0$, and for $t = 1,000$, $5,000$ and $30,000$. As it can be seen, with the growth of t, the standard deviations of the histograms values decrease, leading to the conclusion that with the progress of the simulation, "as time goes by", the effect of the initial randomness decreases. In other words, the long-term outcome of the simulation is well-defined and independent of the actual values in the initial step. Sub-figure E depicts the average of the standard deviations for each time-step t, leading to the same conclusion. These results were calculated for the attitude values towards concept 1, but since the dynamics of the attitude values are governed by the same rules for both concepts (defined by Eq 2 in the main text), these results are valid for concept 2 as well.

Effect of the parameters

The model – as designed to be a minimal model – contains only a couple of parameters, summarized in Table 1 in the main text (repeated hereinafter). In the followings, the effect of their actual values will be inspected.

Notation	Meaning	Values
N	Number of individuals (population size)	100, 1000
T	Number of iterations (level of exposure)	100,000-300,000
Z_A	Noise on the magnitude of attitude change	$\in [-0.01, +0.01]$ (main text),<br>$\in [-0.05, +0.05]$ (SI),<br>$\in [-0.1, +0.1]$ (SI),<br>$\in [-0.2, +0.2]$ (SI),<br>$\in [-0.5, +0.5])$ (SI)
K_O and K_{NB}	Type of the connection between concepts (supportive or conflicting)	$+1$ or -1

Table S1: Summary of the model parameters. Left column: Nomination used in the manuscript. Middle column: description, and right column: values used in the simulation. See also Table 1 in the main text.

Number of agents, N

Equations (1) to (5) (in the main text) govern the dynamics of the attitude values. Since all of these equations include data describing only the focal individual (agent i), individuals develop their attitudes basically independently from each other. This originates from the fact that in the present model, the source of the information does not matter. Accordingly, the exact number of the population (N) has only a statistical effect (as higher number of agents ensures more precise statistics). The results provided in the main text are generated assuming $N = 1000$, but, as Figure S2 demonstrates, the main features of the dynamics are perfectly visible on smaller populations as well (in which $N = 100$). The only way by which the population size matters is the speed of the dynamics, since in smaller populations each agent gets to be selected more frequently – but this feature can be counteracted by longer runs (higher iteration numbers).

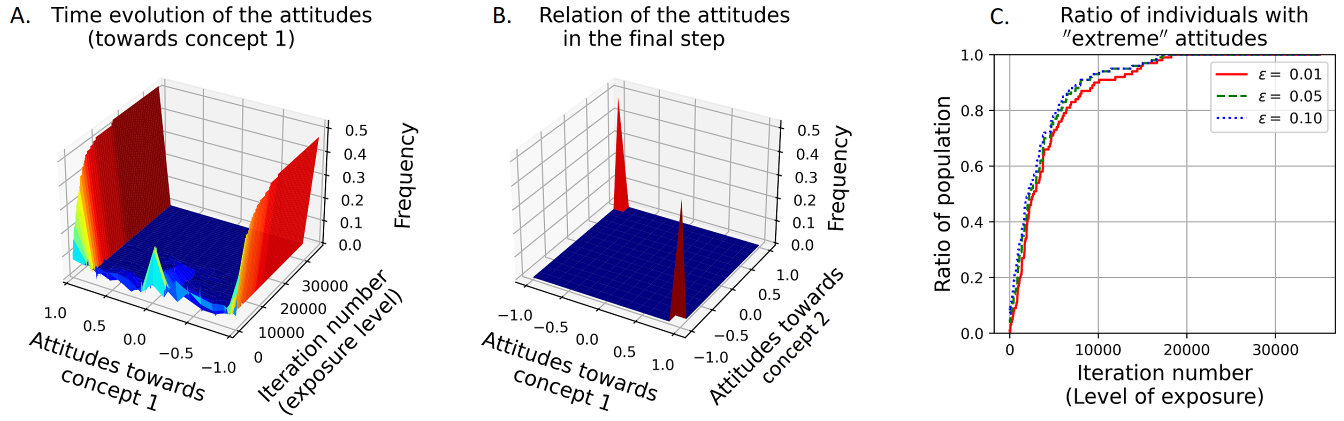

Figure S2: Simulation results for $N = 100$ agents, that is, for one scale smaller population than the one studied in the main text. (A) The evolution of attitudes within a population, due to exposure to a news: similarly to the results reported in the main text, as the level of exposure intensifies – measured by the iteration number t – the attitude values tend towards extremities, marked by $+1$ and -1. At the beginning of the process (at smaller iteration numbers, representing lower exposure) maintaining zero-near attitude values is also a good strategy for avoiding cognitive dissonance. (B) At the end of the simulation, the vast majority of the population adopts extreme attitudes, marked by the sharp peaks at $(+1, -1)$ and $(-1, +1)$. (C) Proportional to the level of exposure (iteration number t), the ratio of population holding extreme attitudes monotonically grows. Parameters: $N = 100$, $Z_A = 0.01$, $T = 35,000$, News connection type: negative ($K_0 = -1$), and finally, a parameter effecting only the visualization: number of bins $= 15$ in sub-figures A and B (instead of the 100, applied for figures in the main text).

Number of iterations (level of exposure), T

As briefly mentioned beforehand, at low level of exposure (expressed by small values of the t iteration number), maintaining neutral attitudes towards one or both concepts also results in the avoidance of cognitive dissonance (See Figure S3). This feature – observed in real-life cases as well – manifests itself in the frequent occurrence of zero-near values (see sub-figure A). However, prolonged exposure to the news/information destroys this neutrality, and pushes the agents towards extremity. The reason behind this phenomenon is that the attitude values immediately start to tend towards the extreme values as the agent tilts towards either direction – in other words, it is an unstable equilibrium point. Accordingly, the higher the noise, the less stable this attitude is (see also Figure S4).

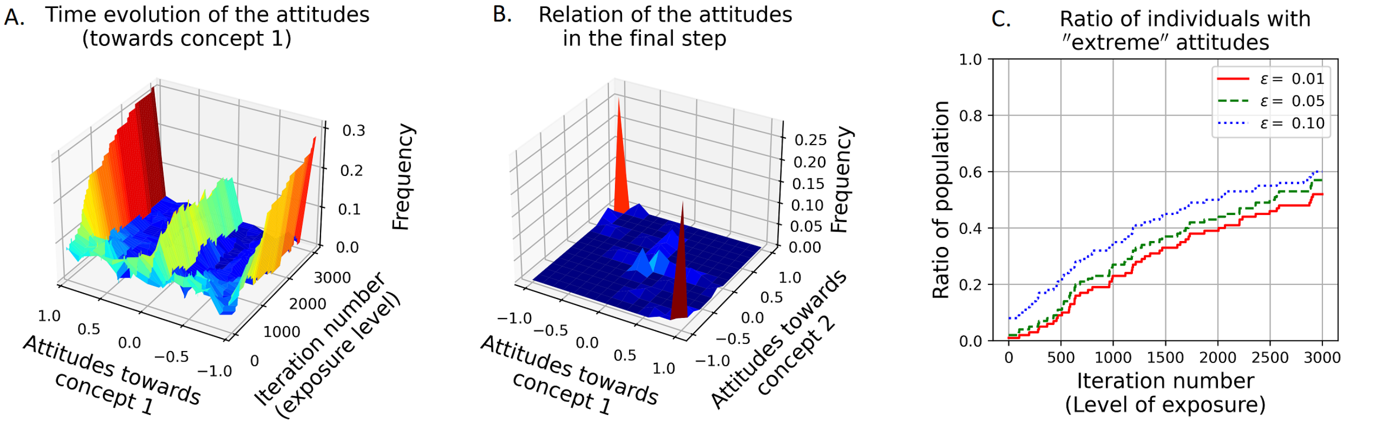

Figure S3: The evolution of attitudes within a population, due to *limited* exposure to news, achieved by limiting the number of iterations in $T = 3000$. (A) In case of limited exposure to news, zero-near values are also common, that is, "centralist" attitudes also appear in high number, since they ensure the avoidance of cognitive dissonance as well. However, since due to the smallest deviation from 0, the attitudes start to tend towards extremities, this is an unstable equilibrium point, and, accordingly, in case of persistent exposure to news and/or experiencing higher levels of noise values, it vanishes, giving rise to extreme stances. (B) While a considerable ratio of the population adopts extreme attitudes (marked by the peaks at $(+1, -1)$ and $(-1, +1)$), individuals also tend to take a neutral stance (characterized by zero-near values) for both concepts. (C) As the exposure to the news intensifies, the ratio of population holding extreme attitudes monotonically grows. Parameters: $N = 100$, $Z_A = 0.01$, $T = 3,000$, News connection type: negative ($K_0 = -1$), and finally, a parameter effecting only the visualization: number of bins = 15 in sub-figures A and B.

Noise, Z_A

Noise is a central element in all social and biological models. In the present approach, two types of noises are included: ρ and Z_A. The fist one, ρ has effect only in case the hierarchical nature of beliefs are also considered (see section "Involving the hierarchical nature of beliefs"). The second one, Z_A defines the maximal value of attitude change (see also Eq (2) in the main text). The bigger the Z_A, the larger is the *magnitude* of the random change of the updated attitude values. This random variation can be either positive or negative, allowing attitudes to change signal. Figure S4 shows the simulation results assuming four different noise levels: $Z_A = 0.05$ (top row), $Z_A = 0.1$ (second row), $Z_A = 0.2$ (third row) and finally, $Z_A = 0.5$ (bottom row). These are to be compared to $Z_A = 0.01$, assumed in the simulations presented in the main text. The most important effect of the noise is the elimination of the neutral stand-points: despite the fact that zero-near attitudes protect the agent from experiencing cognitive dissonance, the higher the noise is, the less stable this equilibrium point is, since noise tilts the attitude values away from 0.

Type of connection, K_O and K_{NB}

In case the way the news connecting the concepts is not negative but positive ($K_0 = +1$ instead of -1), then – according to the expectations – attitudes towards the connected concepts coincide with each other, instead of opposing one an other (see sub-figure B in Figure S5).

This follows from the fact that the level of coherence individual i experiences at time-step t, $C_i(t)$, depends only on the original attitudes, $a_{i,1}(t)$, $a_{i,2}(t)$ and on the connection type K_0, as $C_i(t) = a_{i,1}(t) \cdot a_{i,2}(t) \cdot K_0$ (See also Eq. 1 in the main text). As it can be seen in Figure S5, apart from this effect, the exact choice of K_0 (whether it is +1 or -1) does not have any other effect on the simulation results.

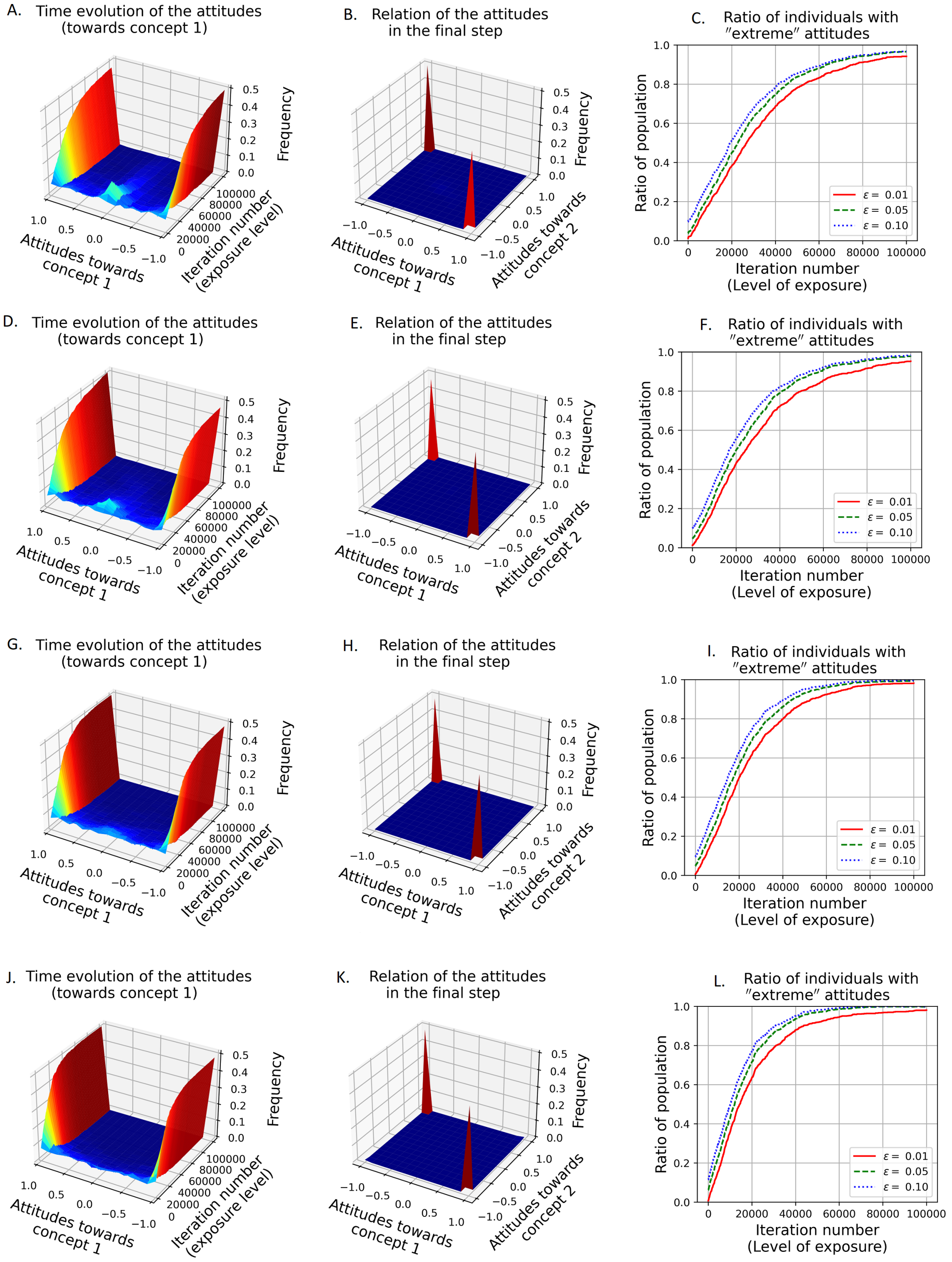

Figure S4: Simulation results with four different noise values: $Z_A = 0.05$ (on sub-figures A to C, top row), $Z_A = 0.1$ (on sub-figures D to F, second row), $Z_A = 0.2$ (on sub-figures G to I, third row) and $Z_A = 0.5$ (on sub-figures J to L, bottom row.) First column: The time evolution of the attitude-histograms; Second column: attitude values in the final step, and third row: ratio of individuals with extreme attitude values. As it can be seen, noise does not effect the long-term outcome of the simulation results, as the attitude values clearly tend towards the (-1, 1) and (1, -1) stable points. However, noise does have an important effect on the short run: the higher the noise is, the less stable the neutral (zero-near) standpoint are, and, accordingly, they vanish more quickly. The parameters are: $N = 1000$, $T = 100,000$, news connection type: negative ($K_0 = -1$), and the number of bins $= 15$

.

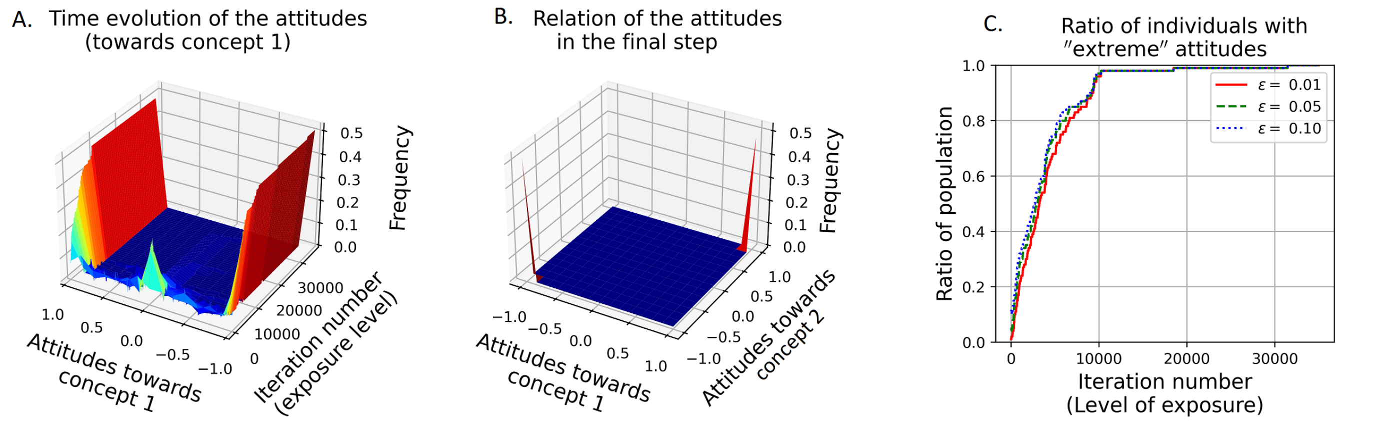

Figure S5: The effect of the choice of K_0, the type of connection. (A) The evolution of attitudes within a population, due to exposure to a news: similarly to the results reported in the main text, as the level of exposure intensifies –measured by the iteration number t – the attitude values tend towards extremities, marked by $+1$ and -1. (B) At the end of the simulation, the vast majority of the population adopts extreme attitudes, *but in this case, they coincide, instead of opposing each other*, marked by the sharp peaks at $(+1, +1)$ and $(-1, -1)$. (C) Proportional to the level of exposure (iteration number t), the ratio of population holding extreme attitudes monotonically grows. Parameters: $N = 100$, $Z_A = 0.01$, $T = 35,000$, News connection type: positive ($K_0 = +1$), and finally, the number of bins = 15 in sub-figures A and B.

The initial distribution of the attitude values

For the results presented in the main text, the initial attitude values (that is: the way agents relate to one or the other concept) are set randomly, according to a uniform distribution, taking values from the $[-1, 1]$ interval. The effect of this stochasticity, originating from the randomly determined initial values, has been analysed in the first part of the Supporting Information, in the section entitled "Statistical behaviour". In the present section simulation results are shown for different settings of initial attitude values. More specifically, four cases are being compared (all taking values from the $[-1, 1]$ interval): (i) uniform distribution, same as in the main text, see Figure S6A (ii) Gauss distribution with $\sigma = 0.4$, (see Figure S6E), (iii) Gauss distribution with $\sigma = 0.2$, (see Figure S6I), and (iv) constant zero (Figure S6M). The central question is that how these initial differences affect the dynamics of the simulation and its long-term outcome? As it can be seen on the third column of Figure S6 (sub-figures C, G, K and O), in all cases, the attitudes tend toward extremities, marked by the sharp peaks at the $[-1, 1]$ and $[1, -1]$ points. At the same time, the dynamics itself is different, as the more close the initial values are to zero, the longer it takes for these neutral standpoints to evolve extreme (see the 2nd column in Figure S6). Note, that in case all initial attribute values were set to be zero (bottom row), the simulation run for 300,000 steps, in contrast with the 150,000, set for the first three cases. At the same time, this process speeds up by applying higher nose levels (see the section entitled "Noise, Z_A", and Figure S4).

Involving the hierarchical nature of beliefs

As mentioned in the main text, human beliefs are organized in a hierarchical manner, meaning that there are more and less central convictions, having bigger or smaller effect on other beliefs. Typically, those that are more close to the *identity* are higher in rank, and, accordingly, more difficult to change.

The robustness of the model results with respect to this consideration has been checked by assuming that more embedded attitudes – that is, those that are more close to +1 or -1 – are more difficult to change. More precisely, the *maximal magnitude* of the change has been set in a way that the more close an attitude is to abs(1), the smaller is the maximal change it can undergo.

Recalling the equation from the main text (Eq. 2), defining the new attitude $a_{i,k}(t+1)$ of agent i at time step $t+1$, due to experiencing a cognitive dissonance (or reassurance) $C_i(t)$, with noise level Z_A:

$$a_{i,k}(t+1) = sign(a_{i,k}(t)) \cdot (\mid a_{i,k}(t) \mid + \rho \cdot C_i(t)) + Z_A \tag{S1}$$

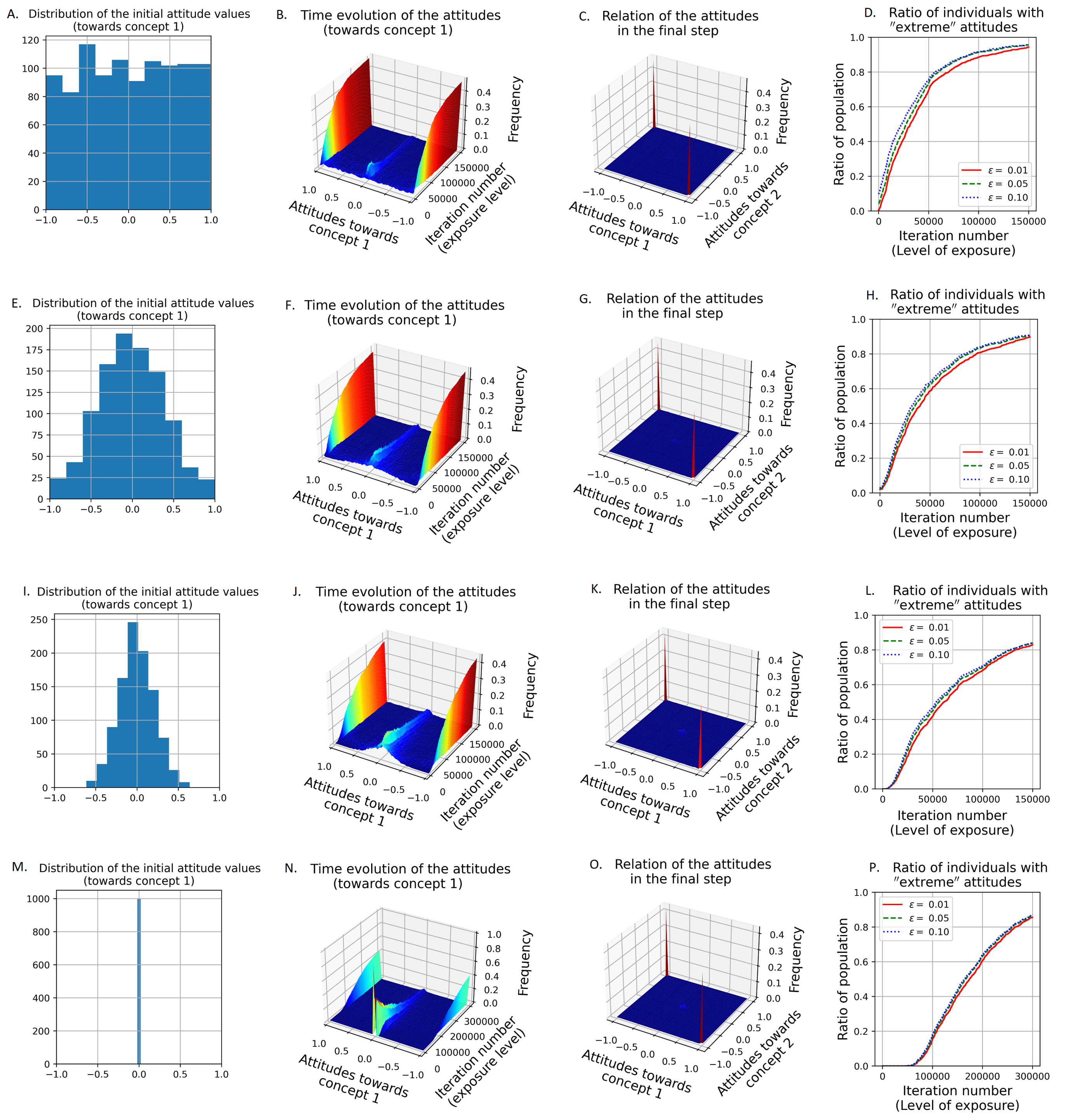

Figure S6: Simulation result with four different initial attitude value distributions: top row: uniform distribution (sub-figure A), 2nd row: Gauss distribution with $\sigma = 0.4$ (sub-figure E), 3rd row: Gauss distribution with $\sigma = 0.2$ (sub-figure I), and bottom row: constant zero distribution (sub-figure M). All simulations tend towards the $[-1, 1]$ and $[1, -1]$ stable points on the long run (3rd column), but the dynamics are different (2nd column), as the more close the initial attitude values are to zero, the longer it takes for these neutral standpoints to evolve into extremities. The parameters are: $N = 1000$, $Z_A = 0.01$, $T = 150,000$ $(300,000)$, News connection type: $K_0 = -1$, and finally, the number of bins $= 50$.

We see that the attitude value changes proportionally to the experienced "coherence" $C_i(t)$ (which is usually referred to as "cognitive dissonance" in case it is negative, and "reassurance", in case it is positive). In the results presented in the main text, ρ is simply a random number taken from the $[0, 1]$ interval with uniform distribution, that is,

$$\rho = R = Rand(0,1)$$

In order to incorporate the hierarchical property of beliefs into the model, ρ has been expanded to have two components: (1) the above random value (taken from the $[0,1]$ interval with uniform distribution), and (2) an "amplitude" value M with which the above random value is multiplied with:

$$\rho = R \cdot M$$

This maximal value M is defined by a simple Gauss function, and adjusted by its standard deviation c in the following way:

$$M = e^{\frac{-a_{i,k}(t)}{c^2}}$$

That is, the smaller the c, the smaller is the maximal attitude change (see also sub-figures A, D and G in Figure S7). For example, in S7A, in case the "(original) attitude towards the concept under modification", $a_{i,k}(t)$, recorded on the horizontal (x) axis, is zero, the maximal change M is 1 (recorded on the vertical (y) axis). But in case $a_{i,k}(t) = abs(0.5)$ then $M \approx 0.67$, if $a_{i,k}(t) = abs(0.75)$ then $M \approx 0.4$, and finally, in case $a_{i,k}(t) \to abs(1)$ then $M \to 0$.

Figure S7 shows the simulation results for 3 different values of c: $c = 0.8$ (top row), $c = 0.5$ (middle row), and $c = 0.3$ (bottom row). The two main observations are that:
(1) as the exposure to the news intensifies (marked by the iteration number t on sub-figures B, E and H), the attitude values tend towards the two extremities, + and -1 (– which is the main claim of the manuscript).
(2) The smaller is the c is (that is, the more pronounced the hierarchical effect), the more are the "centralist" (near-zero) attitudes in case of moderate exposure (small t values).

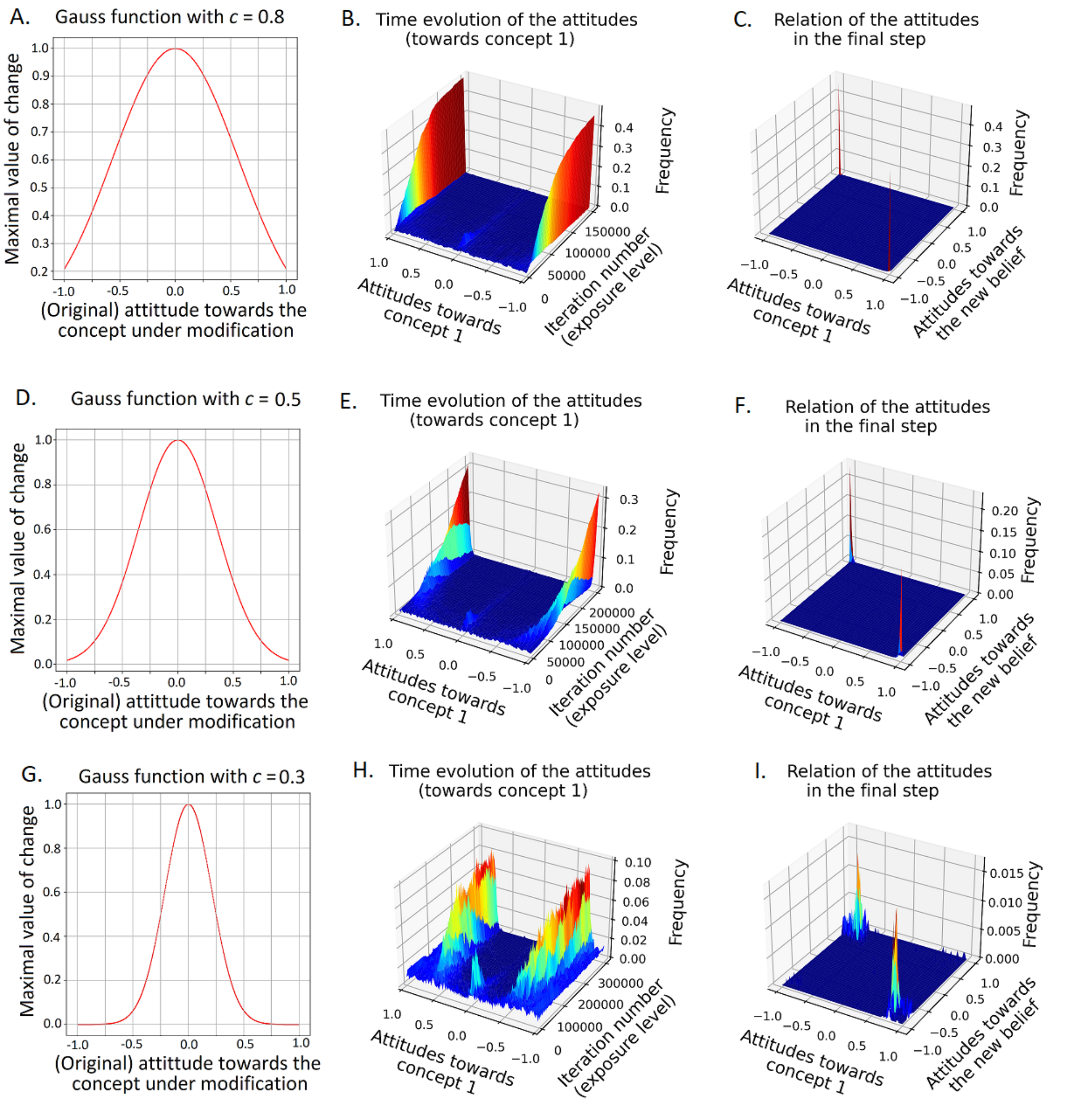

Figure S7: Incorporating the hierarchical nature of beliefs. The change of attitudes within a population due to circulation of news for 3 different values of c: $c = 0.8$ (top row), $c = 0.5$ (middle row), and $c = 0.3$ (bottom row). The smaller the c, the more pronounced is the hierarchical feature of the belief system (that is, the more difficult it is to modify high-ranking beliefs). A, D and G: the maximal value of attitude change M as a function of c. B, E and H: The evolution of one of the attitudes, due to prolonged exposure to the news. The main claim remains unchanged: as the exposure to the news intensifies (marked by the iteration number t), attitude values drift towards the extremities. Furthermore – as a new result – the smaller is the c is (that is, the more pronounced the hierarchical effect), the more are the "centralist" (near-zero) attitudes in case of moderate exposure (small t values). C, F and I: The vast majority of the population adopts extreme attitudes, marked by the peaks at $(-1, +1)$ and $(+1, -1)$, in case of enduring exposure to the news.
